# Daily stress-influence dynamics predict relationship satisfaction in post-stroke couples

**DOI:** 10.3389/fpsyg.2025.1659945

**Published:** 2025-12-04

**Authors:** Sierra Birthelmer, Elizabeth Zambrano Garza, Theresa Pauly, Rachel A. Murphy, Maureen C. Ashe, Kenneth M. Madden, Wolfgang Linden, Denis Gerstorf, Anita DeLongis, Christiane A. Hoppmann

**Affiliations:** 1 Department of Psychology, The University of British Columbia, Vancouver, BC, Canada; 2 Centre for Healthy Aging, The Pennsylvania State University, University Park, PA, United States; 3 Department of Gerontology, Simon Fraser University, Vancouver, BC, Canada; 4 School of Population and Public Health, University of British Columbia, Vancouver, BC, Canada; 5 Cancer Control Research, BC Cancer, Vancouver, BC, Canada; 6 Department of Family Practice, The University of British Columbia, Vancouver, BC, Canada; 7 Department of Medicine, University of British Columbia, Vancouver, BC, Canada; 8 Department of Psychology, Humboldt University, Berlin, Germany

**Keywords:** aging, dyad, relationship satisfaction, stress, influence, stroke, chronic illness

## Abstract

**Introduction:**

Significant health events, such as a stroke, not only impact the individual themselves but also significant others, such as partners. This study examines the daily life associations between stress and relationship satisfaction in couples post-stroke, and the potential buffering effect of perceived equal relationship influence.

**Methods:**

We analyzed data from 85 Canadian couples in whom at least 1 partner experienced a stroke. Both members of the dyads provide simultaneous stress and relationship satisfaction ratings for up to 14 consecutive days. Relationship influence was measured once. We fit three nested two-level actor–partner interdependence models.

**Results:**

Elevated stress was associated with lower same-day relationship satisfaction at both within- and between-person levels. Furthermore, higher daily partner stress was associated with lower actor relationship satisfaction on the same day, over and above one’s daily stress. Finally, an equal relationship influence buffered the negative effects of partner stress on relationship satisfaction for stroke survivors.

**Conclusion:**

This study highlights the complex interplay between daily stress, relationship satisfaction, and relationship influence in couples living with the effects of a stroke. Findings underscore the importance of considering the balance of power in relationships, as stroke survivors and their partners face distinct challenges that shape their daily relational experiences.

## 1 Introduction

A stroke is a debilitating health event that impacts a significant part of the population, with the risk increasing with age (Katan and Luft, 2018). For older adults, partners often serve as the closest relationship and primary source of support following health challenges (Fingerman and Charles, 2010; Hoppmann and Gerstorf, 2016; Lüscher et al., 2022). Consequently, stroke affects not only the survivor but also their partner, who must adapt to new caregiving demands and relationship changes (Berg and Upchurch, 2007). Caring for a partner after a stroke is frequently accompanied by heightened stress and diminished relationship quality (Fox et al., 2021; Godwin et al., 2013; Penning and Wu, 2016; Schulz et al., 2020). However, few studies have examined the day-to-day fluctuations in stress and relationship satisfaction, or the role of relationship influence in moderating these associations.

### 1.1 The influence of partners

Longitudinal research demonstrates that older partners exert mutual influences on each other’s health and wellbeing. Functional decline in one partner can heighten depressive symptoms and reduce activity levels in the other, whereas positive changes tend to be mirrored across the dyad (Fox et al., 2021; Godwin et al., 2013; Hoppmann and Gerstorf, 2016; Levere et al., 2022; Revenson and DeLongis, 2010). When faced with illness or disability, couples must often adapt their routines and shared activities to accommodate new limitations (Berg and Upchurch, 2007). Hence, daily stress levels are often elevated not only for stroke survivors but also for their partners, who frequently shoulder additional physical and emotional caregiving tasks (Godwin et al., 2013; Levere et al., 2022; Revenson et al., 2005).

### 1.2 Chronic illness, daily stress, and relationship satisfaction

Health problems such as stroke can introduce substantial stress into relationships, which in turn contributes to relational strain for both partners. Longitudinal and cross-sectional research indicates that with the onset of a stroke, relationship satisfaction often declines substantially, reflecting the immediate relational strain couples face before long-term adjustment occurs. For example, Godwin et al. (2013) conducted a longitudinal study following stroke survivors and their spousal caregivers over the first-year post-discharge, showing that mutuality declines were associated with higher perceived stress and lower relational wellbeing over time. In contrast, Schulz et al. (2020) summarized cross-sectional evidence demonstrating that family caregiving for older adults, including post-stroke contexts, is frequently accompanied by elevated relational strain and reduced satisfaction (Godwin et al., 2013; Schulz et al., 2020). Providing ongoing physical and emotional support can create persistent psychological and social stress for both members of the couple, leading to lower satisfaction across a range of study designs, including cross-sectional, experimental, longitudinal, and qualitative research with post-acute stroke survivors (Dehghankar et al., 2024; Godwin et al., 2013; Hodson et al., 2020; Schulz et al., 2020).

### 1.3 The role of relationship influence

The substantial adjustments that accompany health challenges such as stroke often prompt shifts in relationship dynamics. A mixed-methods review found that many partners report unequal relationship influence due to an imbalance in their physical and/or cognitive capacities to participate in shared decision-making (Holroyd-Leduc et al., 2016). Such illness-related role changes can disrupt the couple’s usual balance of influence, altering how partners navigate joint responsibilities.

Relationship influence refers to interpersonal sway and is often operationally defined as the extent to which the actions of one person affect the thoughts, feelings, or actions of another person (Oyamot et al., 2010). When influence is imbalanced, couples may experience inequities on who prefers or needs guide important decisions, potentially undermining their relational wellbeing. Longitudinal and cross-sectional studies with young, healthy couples show that perceived imbalance of influence predicts lower relationship satisfaction, and higher attachment insecurity over time (Leonhardt et al., 2020; Traeder and Zeigler-Hill, 2019). In the aftermath of a stroke, maintaining a sense of mutual influence may therefore serve as a key relational resource, helping couples preserve autonomy and stability amid ongoing challenges (Leonhardt et al., 2020; Oyamot et al., 2010).

Couples’ ability to manage stress together plays a critical role in relationship satisfaction and overall wellbeing. The Systemic-Transactional Model of Dyadic Coping (STM; Bodenmann, 1995, 2005) conceptualizes stress as a shared experience that is shaped by interdependent communication processes between partners. According to this model, partners influence one another’s stress appraisals and coping responses through the expression, perception, and regulation of stress. Dyadic coping can take both positive forms, such as empathy, joint problem-solving, or mutual reassurance, and negative forms, such as withdrawal, hostility, or ambivalence, which together shape couples’ emotional adjustment and relationship quality.

Building on this foundation, Berg and Upchurch’s (2007) Developmental-Contextual Model extends the dyadic perspective to chronic illness and aging. This framework emphasizes that couples adapt to ongoing stressors through a renegotiation of roles and responsibilities. Rather than focusing solely on stress communication, the Developmental-Contextual Model situates dyadic coping within a broader developmental and relational context, highlighting how partners’ joint regulation processes are influenced by factors such as age, health, and relationship characteristics. This model is particularly relevant in late-life and illness contexts, where maintaining relational balance and mutual influence can be central to couples’ adjustment.

In the context of stroke, these frameworks collectively underscore that coping requires the coordination of roles and responsibilities. The degree to which both partners perceive a balance of influence in the relationship may therefore reflect how they master these challenges together. Maintaining a sense of mutual influence may serve as a protective relational resource, buffering the effects of daily stress on relationship satisfaction.

### 1.4 The current study

**Figure 1:**
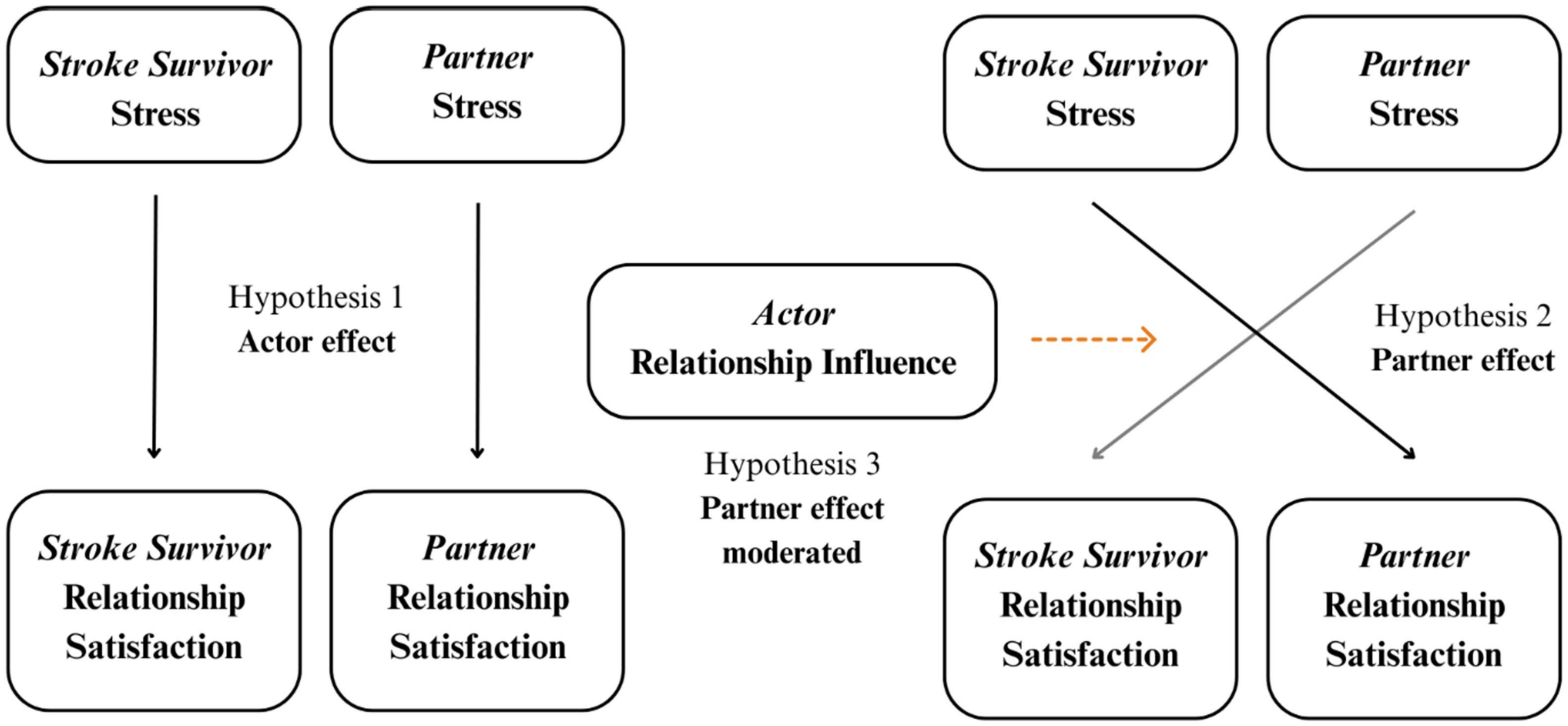
Conceptual model of actor and partner stress effects on relationship satisfaction, moderated by actor relationship influence. ^*^Solid arrows depict the main effects: hypothesis 1 (actor effects)—each person’s own stress (stroke survivor vs. partner) predicts their own relationship satisfaction and hypothesis 2 (partner effect)—each person’s partner’s stress predicts the actor’s relationship satisfaction. The dashed arrow represents the moderating effect of actor relationship influence (hypothesis 3), such that the strength of the negative association between partner stress and actor relationship satisfaction varies as a function of the actor’s perceived influence (equal vs. unequal with their partner).

This brief report extends prior research on stress and relationship satisfaction by examining these processes in daily life among couples at least 3-month post-stroke. Unlike previous cross-sectional studies, the present study uses a dyadic daily-diary design to capture both partners’ experiences over time and to examine how perceived influence moderates these associations. This approach allows us to move beyond general associations toward a better understanding of the moment-to-moment interplay of stress, satisfaction, and influence within couples adjusting to chronic illness. We use data from a repeated daily life assessments study that examined joint health behaviors in couples in which at least one partner is a stroke survivor (Johnson et al., 2025; Luo et al., 2023; Pauly et al., 2021, 2023; Zambrano Garza et al., 2025). Based on previous literature linking stress and relationship satisfaction, we hypothesize that stress and relationship satisfaction will be negatively associated in couples post-stroke, both at the daily and overall levels. Specifically, we hypothesize that individuals post-stroke will report lower relationship satisfaction on days when they experience higher stress. We further expect that partner stress will also predict lower individual relationship satisfaction, beyond the effects of one’s own stress. Additionally, we propose that balanced relationship influence will moderate this partner effect, such that the negative impact of partner stress on relationship satisfaction will be weaker for individuals who report equal influence within their relationship. In our analysis, we control for variables known to be associated with stress, including age, gender, health, and experience of stroke (Almeida et al., 2002, 2011) (see Figure 1).

## 2 Methods

### 2.1 Participants

This study recruited community-dwelling couples made up of individuals post-stroke and their partners. Participants were recruited through community outreach and the registration list of the Heart and Stroke Living with Stroke Program, which was offered biannually at 27 different sites across British Columbia. All data for the study were collected between 2016 and 2019.

In order to partake in the study, couples must have been community-dwelling at the time of participation, and at least one partner of the couple must have previously sustained a stroke. Both partners needed to participate in the study to be eligible. Participants had to have the ability to communicate verbally, read newspaper-sized print, and walk independently, with or without a walking aid, for more than 10 m. Additionally, participants had to be able to provide informed consent. It was also required that there were at least 3 months between participation in the Living with Stroke Program and participation in the study. Participants were not eligible to take part if they were suffering from any medical condition for which their doctor had told them that physical activity or fruit and vegetable consumption was contraindicated. This criterion was included as a precautionary safeguard, as the study focused on health-promoting behaviors that we could not encourage among individuals whose physicians had advised otherwise. No participants were excluded on this basis. Participants self-reported their age, gender, health, and whether or not they had experienced a stroke. For one couple in which both partners had experienced a stroke, the female was assigned the “stroke survivor” role and was given stroke-related questions, whereas her partner was given the “partner” version of the questions to maintain role balance in the study with male stroke survivors.

Of the 244 couples who expressed interest in the original study, 139 met the eligibility criteria. Out of these 139 couples, 101 consented to participate (202 individuals). One couple was a pilot couple; 16 participants (6 couples and 4 individuals) discontinued the study, 13 participants (5 couples and 3 individuals) were excluded due to missing data for the central variables, and 1 participant was excluded due to data fidelity concerns. In total, of the original sample of 202 participants, 32 were removed, leaving 170 individuals in the total sample (85 couples, 82.4% white, 6.88% Asian, 0.55% Black, 1.44% Indigenous, 0.89% Hispanic, 3.46% Mixed race, 2.87% other, *M*_age_ = 66.95, SD_age_ = 9.75, range = 33–88, 50% women, 50% men).

### 2.2 Procedure

Eligible participants who provided written informed consent were asked to participate in a 3-h baseline session, a 14-day time-sampling phase, a blood draw, and an exit session, as well as participant-driven iPad mini workshops. All study material was administered through Qualtrics. Each couple received two iPad minis to be able to complete the necessary materials for the study. As compensation for participation, each couple was offered to keep one tablet or receive $100 CAD each. The University of British Columbia Clinical Research Ethics Board reviewed and approved the study (H16-01189-A028). The hypotheses in this study were preregistered on OSF (osf.io/nk4fs). Only measures relevant to the present study will be described.

At the beginning of the study, participants were asked to complete an in-person baseline session. During this session, couples were provided with demographic information and completed socio-motivational questionnaires and health measures. The day after the baseline was completed, couples started the 14-day time-sampling phase with two daily assessments: one right after getting up in the morning and one before going to bed. After the time-sampling phase, participants returned their materials in person and completed an exit session.

### 2.3 Measurements

The present analyses focus on two core variables assessed during the daily diary phase: daily stress, daily relationship satisfaction, and relationship influence.

#### 2.3.1 Daily stress

In the evening, participants were asked to answer the question, *“How stressed did you feel today?”* on a scale from 0 (not at all) to 100 (very much; *M* = 25.65, SD = 28.08).

#### 2.3.2 Daily relationship satisfaction

To assess daily feelings of relationship satisfaction, participants were asked, *“How satisfied were you with your relationship with your partner today?”* during the evening questionnaire. Answers ranged on a scale from 0 (not at all) to 100 (very much; *M* = 80.83, SD = 20.05).

#### 2.3.3 Relationship influence

During the exit session, participants were asked to rate how much “say” they felt they had in their relationship using the *influence meter* (Oyamot et al., 2010). The influence meter is rated on a scale from 1 to 5, with 1 representing the actor having the most influence and 5 representing the partner having the most influence. A binary measure was used to examine the moderation of the influence meter on the effects of stress on relationship satisfaction, with 1 indicating equal influence between partners (rating of 3 on the influence meter) and 0 indicating all other ratings (*M* = 3.11, SD = 0.79).

### 2.4 Covariates

Covariates included gender (men = 1, women = 0), age (in years; grand-mean centered), self-rated health, and stroke status (stroke = 1, no stroke = 0). Self-rated health was measured with the item, *“How would you rate your overall health at the present time?,”* rated on a 5-point scale from 0 (*poor*) to 4 (*excellent*). Missing values for age and health were replaced with the sample mean prior to analysis.

### 2.5 Data analysis

Analyses were performed using the lme4 package in R (Bates et al., 2015), implementing the actor–partner interdependence model (APIM; Bolger et al., 2000) to examine associations between stress and relationship satisfaction. We fit three nested two-level actor–partner interdependence models in nlme:lme. Daily diary reports (level 1, days) were nested within couples (level 2, dyads). All day-level predictors were person-mean centered, and between-person covariates (age, gender, self-rated health, stroke status) were grand-mean centered. Random intercepts accounted for non-independence of repeated measures, and residuals were allowed to differ by stroke status.

#### 2.5.1 Model 1—Actor-stress model

Actor-reported daily stress predicted same-day actor relationship satisfaction for stroke survivor and their partner.

#### 2.5.2 Model 2—Partner-stress model

Partner-reported daily stress predicts actor relationship satisfaction, controlling for actor stress for the stroke survivor and their partner.

#### 2.5.3 Model 3—Moderated partner-stress model

We added a cross-level interaction between partner stress and actor relationship satisfaction by actor-perceived influence to test whether balanced decision power attenuates the partner-stress effect. Significant interactions were probed with simple slope analyses at influence = 0 and 1. We report unstandardized estimates with 95% confidence intervals.

## 3 Results

### 3.1 Descriptive analyses

Given the study’s focus on couples post-stroke, we compared stroke survivors and their partners across key study variables to better understand potential role differences in stress, relationship satisfaction, and perceived influence. Descriptive statistics and bivariate correlations are presented in Supplementary Tables 1a,b. Among stroke survivors, relationship satisfaction was positively associated with age and male gender, and negatively associated with education and both morning and evening stress. Morning and evening stress were moderately correlated. For partners, relationship satisfaction was positively associated with age and self-rated health, and negatively associated with female gender, education, and both morning and evening stress. Morning and evening stress were strongly correlated with partners as well. These descriptive patterns align with prior studies showing that greater stress is generally linked to lower relationship satisfaction, whereas better health and older age are associated with higher satisfaction in older adult couples.

### 3.2 Chronic illness, stress, and relationship satisfaction—the influence of partners

To evaluate our hypotheses that daily stress, both actor and partner, and relationship satisfaction would be negatively associated, we conducted multilevel regression analyses using evening questionnaire data (see Table 1).

**Table 1:** Multilevel actor–partner models predicting daily relationship satisfaction from actor stress, partner stress, and perceived influence in stroke-survivor/partner couples (170 participants, 85 couples).

Stroke survivor/partner									
	Model 1			Model 2			Model 3		
Predictors	Estimates	CI	*p*	Estimates	CI	*p*	Estimates	CI	*p*
Intercept	73.84/87.35	64.42, 85.27/81.38, 93.33	**<0.001/<0.001**	81.49/91.34	70.04, 92.93/85.23, 97.45	**<0.001/<0.001**	75.48/80.22	58.03, 92.93/64.14, 96.29	**<0.001/<0.001**
Influence	–	–	–	–	–	–	−0.04/5.75	−6.72, 6.64/0.25, 11.25	0.990/**0.041**
Actor stress (WP)	−0.06/−0.06	−0.10, 0.01/−0.11, −0.01	**0.015/0.011**	−0.04/−0.05	−0.09, 0.00/ −0.10, −0.01	**0.049/0.021**	−0.06/−0.06	−0.11, −0.01/ −0.11, 0.00	**0.023/0.033**
Actor stress (BP)	−0.13/−0.07	−0.31, 0.05/−0.19, 0.05	0.151/0.264	−0.10/−0.05	−0.29, 0.09/−0.18, 0.08	0.314/0.475	−0.10/−0.02	−0.29, 0.10/ −0.19, 0.14	0.345/0.771
Partner stress (WP)	–	–	–	−0.14/−0.08	−0.19, −0.10/−0.13, −0.04	**<0.001/<0.001**	−0.36/–0.18	−0.57, −0.14/−0.35, −0.02	**0.001/0.031**
Partner Stress (BP)	–	–	–	−0.23/−0.23	−0.38, −0.08/−0.39, 0.07	**0.003/0.006**	−0.16/−0.20	−0.33, 0.01/−0.39, −0.01	0.060/**0.037**
Partner stress X actor influence (WP)	–	–	–	–	–	–	0.14/0.06	0.02, 0.26/−0.04, 0.16	**0.018**/0.231
Age	0.04/0.18	−0.28, 0.37/−0.10, 0.45	0.786/0.214	0.00/0.14	−0.31, 0.31/−0.13, 0.40	0.993/0.308	0.01/0.12	−0.31, 0.32/−0.18, 0.41	0.964/0.445
Gender	12.09/−5.06	4.56, 19.61/−11.50, 1.37	**0.002/**0.123	9.46/−4.17	2.19, 16.72/−10.35, 2.00	**0.011**/0.185	8.35/−4.19	0.93, 15.76/−10.89, 2.51	**0.027**/0.220
Health (stroke survivor)	2.47	−1.04, 5.99	0.167	2.03	−1.29, 5.35	0.231	0.16	−3.32, 3.63	0.930
Random effects									
Residual variance	200.12			194.80			202.03		
Random-slope variance − stress	143.02			129.99			375.66		
Random-slope variance − influence × stroke	–			–			48.23		
Random-slope variance − influence × partner	–			–			2.20		
Intercept–slope correlation	0.32			0.33			0.74		
	–			–			−0.63		
	–			–			−0.10		
ICC	0.46			0.44			0.41		
*N*_Couples_	85			85			71		
Observations	1976			1976			1,652		
Marginal *R*^2^/conditional *R*^2^	0.074/0.502			0.144/0.522			0.133/0.491		

WP, within-person; BP, between-person. Gender was coded as men = 1, women = 0. Stroke status was coded as stroke = 1 and no stroke = 0. All continuous predictors were grand-mean centered. Estimates are unstandardized. *p* < 0.05 are printed in bold. ICCs ranged from 0.41 to 0.46, indicating that 41–46% of the variance was at the couple level and 54–59% at the day level.

**Figure 2:**
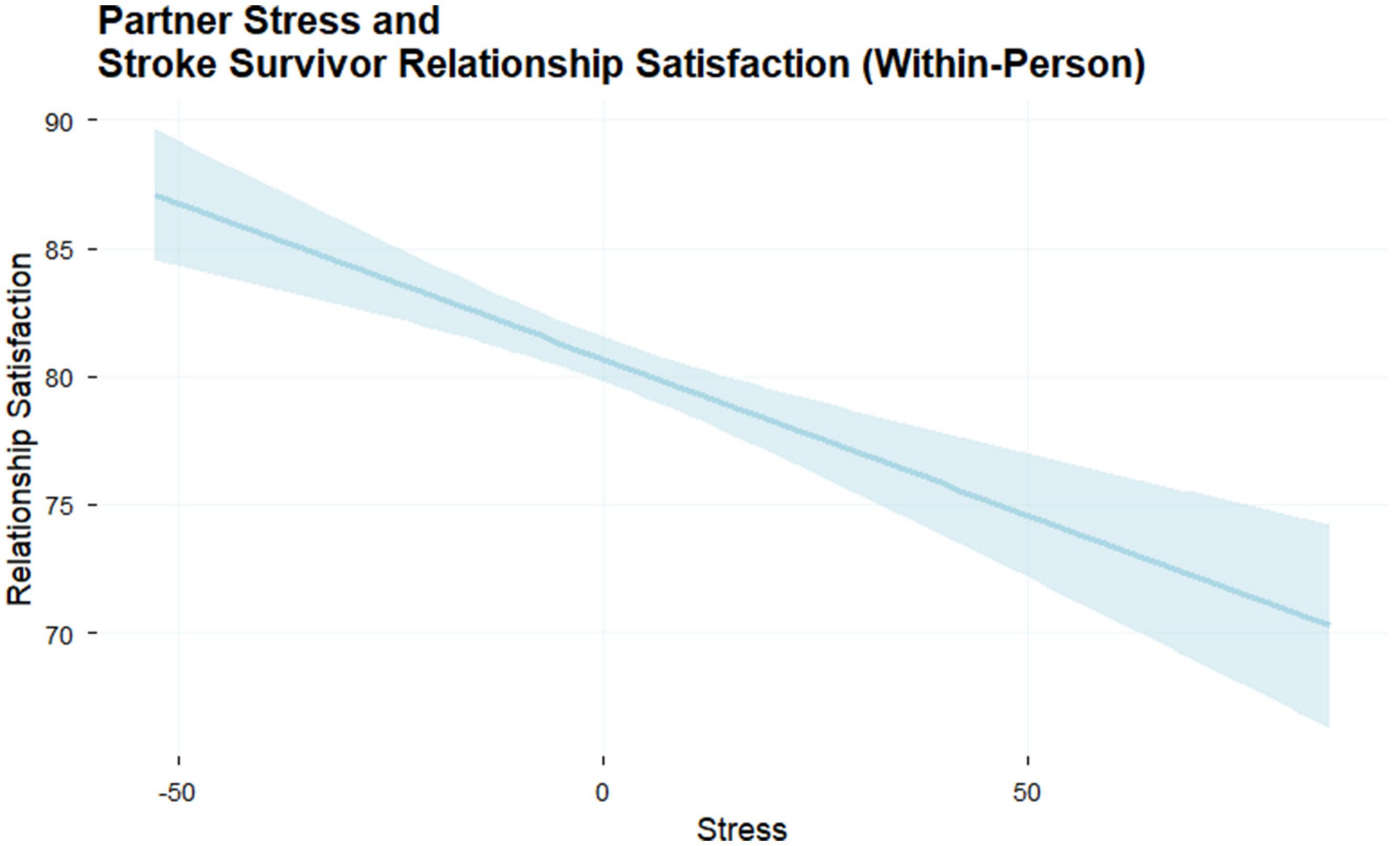
Daily partner associations for evening stress and relationship satisfaction. This figure illustrates the association between partner evening stress and stroke survivor relationship satisfaction. When partner stress was higher, stroke survivor relationship satisfaction was lower. Similar results were seen for stroke survivor stress and partner relationship satisfaction. Shaded areas represent 95% confidence intervals.

Results demonstrate that on days when actors reported more stress, relationship satisfaction was lower. This was true for both stroke survivors (*b* = −0.06, *p* = 0.015) and their partners (*b* = −0.06, *p* = 0.011; Table 1, model 1). To test hypothesis 2, we examined the associations between partner stress and actor relationship satisfaction at the within- and between-person levels. At the between-person level, greater overall partner stress predicted lower actor relationship satisfaction for stroke survivors (*b* = −0.23, *p* = 0.003) and partners (*b* = −0.23, *p* = 0.006). At the within-person level, higher partner stress was associated with lower same-day relationship satisfaction for stroke survivors (*b* = −0.14, *p* = <0.001) and their partners (*b* = −0.08, *p* = <0.001), over and above the actor’s own stress effects (see Table 1, model 2; Figure 2).

### 3.3 The role of relationship influence

**Figure 3:**
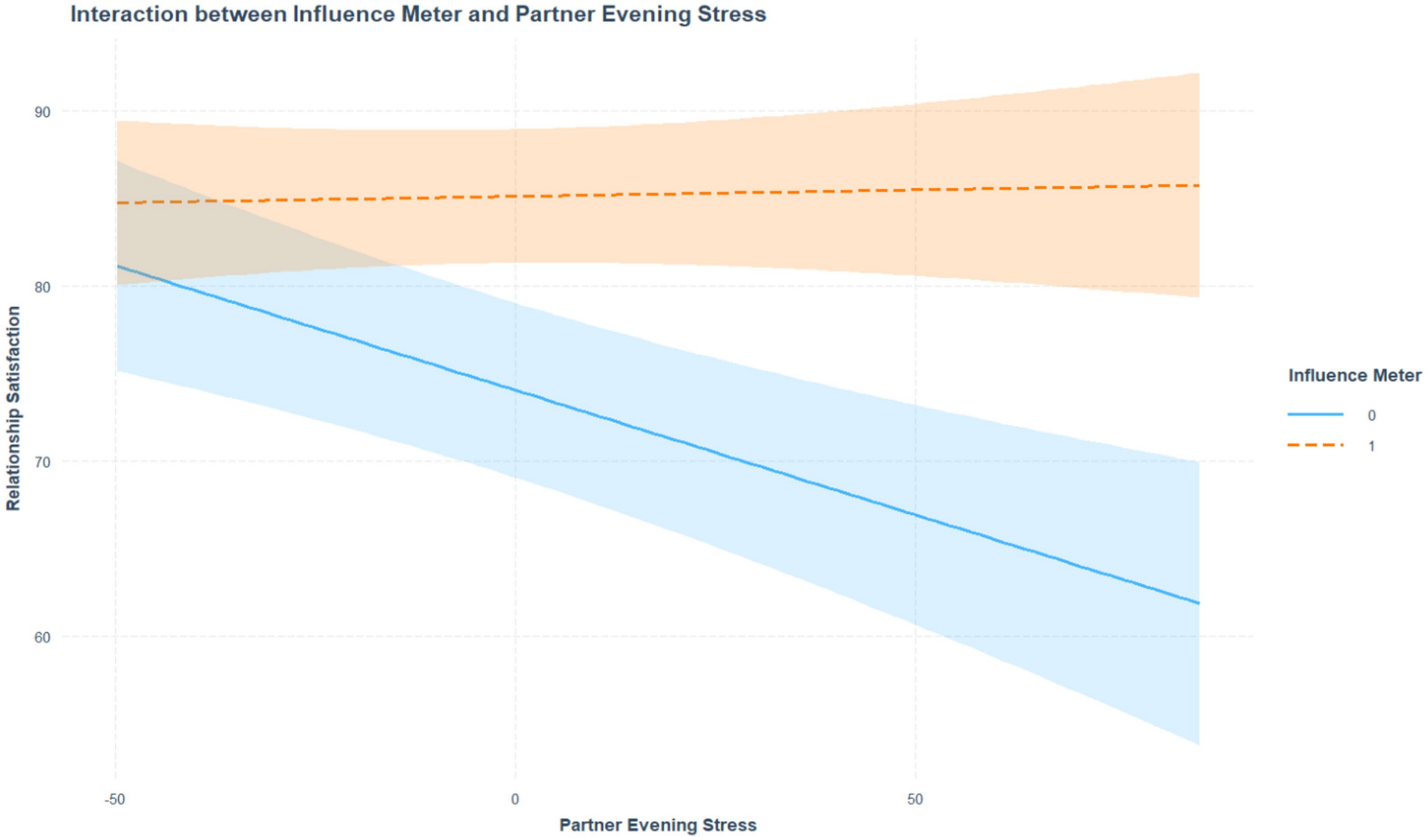
Daily associations for evening stress and relationship satisfaction*influence meter—actor stroke. This figure illustrates the interaction between partner evening stress and stroke survivor influence meter scores on own relationship satisfaction. When the influence meter score was 1 (equal), the negative association between partner stress and stroke survivor relationship satisfaction was weaker. When influence was rated as unequal (0), this association was attenuated, suggesting that equal influence in the relationship may buffer the negative effects of partner stress on relationship satisfaction for stroke survivors. Shaded areas represent 95% confidence intervals.

To test hypothesis 3, we conducted moderation analyses examining whether the actor relationship influence buffered the predictive effect of partner stress for actor relationship satisfaction. A significant interaction emerged for stroke survivors: on days when partner stress was high, stroke survivors who reported equal influence did not report lower relationship satisfaction (*b* = 0.14, *p* = 0.018; Table 1, model 3; Figure 3). This buffering effect was not observed among partners.

## 4 Discussion

Consistent with predictions, daily stress was linked to lower daily relationship satisfaction at both the within-person actor level and the between-person and within-person partner level. Notably, perceiving equal decision-making influence buffered this association only for stroke survivors, suggesting that perceiving an equal influence in the relationship, even amid changing roles, may buffer against the relational strain introduced by a partner’s stress. This inter- and intra-individual variability underscores the value of a daily-diary approach to capturing within-couple dynamics.

### 4.1 Stress and relationship satisfaction by partners

The findings of this study indicate that higher own and partner daily stress are consistently associated with lower relationship satisfaction. These findings align with the stroke literature, showing that chronic illness introduces daily strain into close relationships, often resulting in reduced relationship satisfaction for both partners (Fox et al., 2021; Godwin et al., 2013; Schulz et al., 2020). The experience of stroke brings not only physical limitations but also psychological and emotional challenges that affect both the individual and their partner (Berg and Upchurch, 2007; Hodson et al., 2020; Penning and Wu, 2016). Our findings support this assumption by demonstrating that daily stress, whether experienced by oneself or by a partner, can erode daily relationship satisfaction, highlighting the importance of understanding relational wellbeing as a shared, interdependent experience in the context of chronic illness. Furthermore, this study builds on past studies by examining these dynamics in everyday life, adding insights into how stroke-related stress plays out daily within couples.

### 4.2 The role of relationship influence

The findings of this study also align with our moderation hypothesis in that equal decision-making buffered the partner-stress effects, but only for stroke survivors. One possible explanation is that stroke survivors, whose autonomy and daily functioning may be compromised by their health, derive particular benefit from perceiving an equal say in their relationship, whereas their partners’ attention to caregiving demands may attenuate the salience of perceived influence as a protective factor.

### 4.3 Study limitations and future directions

On closing, we note the limitations of our study sample, measures, and design. Since the sample consisted of community-dwelling individuals, stroke severity was likely lower than in hospital-based or inpatient rehabilitation samples. Relationship satisfaction scores were also high on average, and reported stress levels were relatively low, potentially reducing our ability to detect stronger associations. Most participants perceived equal influence in their relationships (69%), limiting the variability needed to fully examine the moderating role.

As to the limitations of our study design, we note that we only had 14 days of evening diaries. Future studies could include more time periods and more questionnaires per day to better capture day-to-day fluctuations in stress and relationship satisfaction. Future research should also aim to include samples with greater variability in health status, relationship dynamics, and cultural backgrounds to better understand for whom and under what conditions equal influence moderates stress.

Another avenue for future research concerns how help is communicated. Support-visibility theories distinguish invisible support, subtle, autonomy-preserving aid shown to help generally satisfied community couples (Bolger et al., 2000), from Gottman’s theory of emotional bids, which focuses on maritally distressed couples, suggesting that overt, responsive support is especially important (Lebow et al., 2019). Our sample occupies a middle ground, as they might not be maritally distressed but do face more daily stress due to the demands of chronic illness. Future studies should test whether the benefits of balanced influence depend on the form in which support is communicated. Such studies could clarify whether overt or more subtle forms of assistance best sustain relationship satisfaction under daily stress.

## 5 Conclusion

This study highlights the complex interplay between stress, relationship satisfaction, and relationship influence in couples living with the effects of a stroke. The findings underscore the importance of considering role-specific dynamics, as stroke survivors and their partners face distinct challenges that shape their daily relational experiences. By capturing both individual and partner effects, as well as the moderating role of influence, this study contributes to a more nuanced understanding of the impact of chronic illness on relationships. These insights emphasize the need for comprehensive, relationship-sensitive support systems that address both partners’ needs and foster balance in couples navigating chronic illness.

## Data Availability

The data analyzed in this study is subject to the following licenses/restrictions: data for this study cannot be openly shared due to containing information that could compromise research participants’ privacy and consent. This concern is particularly heightened with family data, where the ability to identify one’s own data may inadvertently expose the privacy of other family or dyad members (Stine-Morrow and Manavbasi, 2022). Requests to access these datasets should be directed to Christiane Hoppmann, choppmann@psych.ubc.ca.

## Author’s note

This manuscript is based on data and analyses included in the author’s Master’s thesis completed at the University of British Columbia.
