# Supplementary material for: Daily stress-influence dynamics predict relationship satisfaction in post-stroke couples
Source: Front Psychol. 2025 Dec 4;16:1659945. doi: 10.3389/fpsyg.2025.1659945 (PMC12713534; doi:10.3389/fpsyg.2025.1659945)
Supplement: Supplementary file 1 [file Table_1.docx]

**Table 1a**

**Stroke Survivor -** *Means, standard deviations, and correlations with confidence intervals*

| Variable | *M* | *SD* | 1 | 2 | 3 | 4 | 5 | 6 | 7 |
| --- | --- | --- | --- | --- | --- | --- | --- | --- | --- |
|  |  |  |  |  |  |  |  |  |  |
| 1. Relationship Satisfaction | 81.83 | 18.62 |  |  |  |  |  |  |  |
|  |  |  |  |  |  |  |  |  |  |
| 2. Age | 68.46 | 9.98 | .16** |  |  |  |  |  |  |
|  |  |  | [.10, .21] |  |  |  |  |  |  |
|  |  |  |  |  |  |  |  |  |  |
| 3. Gender | 0.24 | 0.43 | .15** | -.14** |  |  |  |  |  |
|  |  |  | [.10, .21] | [-.19, -.08] |  |  |  |  |  |
|  |  |  |  |  |  |  |  |  |  |
| 4. Health | 2.54 | 0.89 | .03 | .17** | -.03 |  |  |  |  |
|  |  |  | [-.03, .08] | [.12, .23] | [-.09, .03] |  |  |  |  |
|  |  |  |  |  |  |  |  |  |  |
| 5. Education | 0.54 | 0.50 | -.16** | .04 | -.27** | .20** |  |  |  |
|  |  |  | [-.22, -.11] | [-.02, .10] | [-.32, -.21] | [.15, .26] |  |  |  |
|  |  |  |  |  |  |  |  |  |  |
| 6. Morning Stress | 21.37 | 24.87 | -.19** | -.08** | -.03 | -.13** | -.02 |  |  |
|  |  |  | [-.25, -.13] | [-.14, -.02] | [-.09, .03] | [-.19, -.07] | [-.08, .04] |  |  |
|  |  |  |  |  |  |  |  |  |  |
| 7. Evening Stress | 23.01 | 26.22 | -.25** | -.12** | -.08* | -.18** | -.03 | .50** |  |
|  |  |  | [-.31, -.20] | [-.18, -.06] | [-.14, -.02] | [-.23, -.12] | [-.09, .03] | [.45, .55] |  |
|  |  |  |  |  |  |  |  |  |  |
| 8. Influence | 1.74 | 0.44 | .16 | .22* | .16 | .03 | -.03 | -.16 | -.09 |
|  |  |  | [-.07, .37] | [.00, .42] | [-.06, .37] | [-.19, .25] | [-.24, .20] | [-.38, .07] | [-.32, .14] |
|  |  |  |  |  |  |  |  |  |  |

*Note.* *M* and *SD* are used to represent mean and standard deviation, respectively. Values in square brackets indicate the 95% confidence interval for each correlation. The confidence interval is a plausible range of population correlations that could have caused the sample correlation (Cumming, 2014). * indicates *p* < .05. ** indicates *p* < .01.

**Table 1b**

**Partner -** *Means, standard deviations, and correlations with confidence intervals*

| Variable | *M* | *SD* | 1 | 2 | 3 | 4 | 5 | 6 | 7 |
| --- | --- | --- | --- | --- | --- | --- | --- | --- | --- |
|  |  |  |  |  |  |  |  |  |  |
| 1. Relationship Satisfaction | 79.86 | 21.32 |  |  |  |  |  |  |  |
|  |  |  |  |  |  |  |  |  |  |
| 2. Age | 66.06 | 9.53 | .07* |  |  |  |  |  |  |
|  |  |  | [.01, .12] |  |  |  |  |  |  |
|  |  |  |  |  |  |  |  |  |  |
| 3. Gender | 0.76 | 0.43 | -.28** | -.09** |  |  |  |  |  |
|  |  |  | [-.33, -.22] | [-.14, -.03] |  |  |  |  |  |
|  |  |  |  |  |  |  |  |  |  |
| 4. Health | 3.21 | 0.85 | .13** | .02 | -.03 |  |  |  |  |
|  |  |  | [.07, .18] | [-.04, .07] | [-.08, .03] |  |  |  |  |
|  |  |  |  |  |  |  |  |  |  |
| 5. Education | 0.47 | 0.50 | -.09** | -.15** | .10** | .14** |  |  |  |
|  |  |  | [-.15, -.03] | [-.21, -.10] | [.04, .16] | [.08, .20] |  |  |  |
|  |  |  |  |  |  |  |  |  |  |
| 6. Morning Stress | 26.93 | 30.17 | -.29** | -.11** | .16** | -.02 | .14** |  |  |
|  |  |  | [-.35, -.23] | [-.17, -.05] | [.10, .22] | [-.08, .04] | [.08, .20] |  |  |
|  |  |  |  |  |  |  |  |  |  |
| 7. Evening Stress | 28.22 | 29.57 | -.39** | -.13** | .17** | -.06* | .10** | .67** |  |
|  |  |  | [-.44, -.34] | [-.18, -.07] | [.11, .23] | [-.12, -.00] | [.04, .15] | [.64, .70] |  |
|  |  |  |  |  |  |  |  |  |  |
| 8. Influence | 1.64 | 0.48 | .34** | .13 | -.15 | .14 | .01 | -.10 | -.13 |
|  |  |  | [.14, .52] | [-.09, .33] | [-.35, .07] | [-.08, .34] | [-.21, .22] | [-.32, .12] | [-.34, .09] |
|  |  |  |  |  |  |  |  |  |  |

*Note.* *M* and *SD* are used to represent mean and standard deviation, respectively. Values in square brackets indicate the 95% confidence interval for each correlation. The confidence interval is a plausible range of population correlations that could have caused the sample correlation (Cumming, 2014). * indicates *p* < .05. ** indicates *p* < .01.
